# Commonly cited approaches to reducing health inequalities: a call for more clarity around their definition and underlying assumptions

**DOI:** 10.1136/jech-2025-224952

**Published:** 2025-12-09

**Authors:** Mhairi Campbell, Bryony Dawkins, Gillian Fergie, Anne-Sophie Jung, Ruth Lewis, Lisa McDaid, Jonathan R Olsen, Roxana Pollack, Benjamin P Rigby, Mark Robinson, Kathryn Skivington, Michael Thomson, Anna Pearce

**Affiliations:** 1 School of Health and Wellbeing, University of Glasgow, Glasgow, UK; 2 Academic Unit of Health Economics, University of Leeds, Leeds, England, UK; 3 School of Politics and International Studies, University of Leeds, Leeds, England, UK; 4 Institute for Social Science Research, The University of Queensland, Brisbane, Queensland, Australia; 5 Population Health Sciences Institute, Newcastle University, Newcastle upon Tyne, England, UK; 6 Institute for Social Science Research, The University of Queensland, Saint Lucia, Queensland, Australia; 7 School of Law, University of Technology Sydney, Sydney, New South Wales, Australia; 8 School of Law, University of Leeds, Leeds, England, UK

**Keywords:** Health inequalities, PUBLIC HEALTH, POLICY

## Abstract

Addressing health inequalities is an international priority. Various approaches have gained popularity in the academic literature and policy-making documents. However, there has been a lack of progress in tackling health inequalities. We outline the main characteristics and principles of five commonly cited approaches: asset-based, place-based, upstream, systems-based and proportionate universalism. We examine how these approaches are described in the literature and the logic by which they are thought to tackle health inequalities.

There was variation in how each approach was described and interpreted. The logic behind how the approaches could improve population health was clearly articulated but often under-developed with respect to health inequalities. Although rarely acknowledged explicitly, it was implied that these approaches seek to reduce health inequalities through focussing on more socially disadvantaged sub-groups in a population, identifying the most impactful intervention levers and/or working to minimise stigma and minimise inequalities in access and uptake of interventions. More attention should be paid to the important principles, features and underlying logic of these approaches in relation to health inequalities to better understand the potential supports and barriers to their success. This will support those working to implement them to do so in ways that are sensitive to local and contextual specificities.

**WHAT IS ALREADY KNOWN ON THIS TOPIC**

- Several approaches to conceptualising the design of interventions and policies to tackle health inequalities have emerged. However, there is a lack of clarity in how these approaches are operationalised.

**WHAT THIS STUDY ADDS**

- Researchers, practitioners and policy-makers are provided with a foundation to facilitate a shared understanding of common approaches to reducing health inequalities.

**HOW THIS STUDY MIGHT AFFECT RESEARCH, PRACTICE OR POLICY**

- A clearer understanding and articulation of approaches that are widely thought to contribute to the reduction of health inequalities will help to identify the potential supports and barriers to their success and guide future actions.

## Introduction

 Health inequalities refer to the ‘systematic, avoidable, and unfair differences in health that can be observed between populations, between social groups within the same population or as a social gradient across a population ranked by social position’.^1^^, page 22^ Social groups can be defined according to socio-demographic characteristics (eg, age, gender, ethnicity, geography of residence), socio-economic characteristics (eg, income, education, neighbourhood deprivation levels) and protected characteristics, including disability and sexual orientation.^2^ The need to tackle health inequalities, and how to go about doing this, has been the focus of scientific scrutiny, and national and international policy agendas for decades.^3^

Several typologies have been proposed to categorise actions to reduce health inequalities, often (but not always) with socio-economic health inequalities in mind. Whitehead^4^ proposed categories of actions, based on distinct aims: strengthening individuals, strengthening communities, improving living and working conditions and related essential services and promoting healthy macro policies. Graham considered where in the population intended benefits are directed—health improvements in the most disadvantaged socio-economic positions (regardless of health changes in other groups), narrowing gaps between those in the least and most advantaged socio-economic groups or reducing health inequalities throughout the whole population (to reduce the social gradient in health).^5^ Benach adapted Graham’s typology to consider in more detail how different targeting approaches can shift population health and impact health inequalities.^6^ Diderichsen and Whitehead identified the mechanisms through which policies could alter health inequalities: reducing social inequalities (or aiming to equalise social position), reducing the higher health-harming risk factors experienced by less advantaged groups, decreasing the impacts of risk factors on health and minimising the consequences of ill health on future social position.^7^ Davey *et al* produced overarching guiding principles for reducing geographical health inequalities which span the most promising high-level foci for interventions/policies (health supporting, easy to use), levels of implementation and timeframe (long-term, multi-sector), the design process (locally tailored) and methods of targeting (eg, matching resources to need, with targeting at disadvantaged communities).^8^

Since the publication of these seminal papers, several approaches for designing interventions and policies to tackle inequalities have gained prominence: asset-based, place-based and systems-based approaches, those which focus ‘upstream’ drivers of inequalities, and proportionate universalism. While they do not neatly fit into a single typology, they have been selected for their frequent citations in the public health, epidemiology, social policy and geography literature and increasing popularity in policy documents.^9^ There has been a disappointing lack of progress in tackling health inequalities, despite decades of concerted efforts.^10^,^13^ A clearer understanding and articulation of approaches that are widely thought to contribute to the reduction of health inequalities will help to identify the potential supports and barriers to their success and guide future actions.^9 14^ We therefore outline how these approaches are described with respect to their definitions and the logic behind the proposed potential for reducing health inequalities. In doing so, we hope to provide a foundation from which to build a shared understanding among researchers, practitioners and policy-makers of the principles behind what they are trying to achieve, and how.

## Commonly cited approaches for reducing health inequalities

The descriptions of these approaches have been drawn from work already known to the authors who have experience of carrying out research in the field of population health and health inequalities in the UK and Australia, supplemented by purposive searches. This stemmed from a 2-day workshop during which experiences and knowledge of the challenges facing UK and Australian policy-makers were shared. Through these discussions, approaches were identified as terms frequently referred to in policy and research. Subsequent meetings involved iterative formulation of the scope of this paper. Understanding of the selected approaches was then supplemented by literature searches on each selected topic, the results of the searches were scanned and relevant information used to develop relevant sections. Table 1 provides examples of interventions or policies that have taken these different approaches (as described by the original authors). Table 2 provides a summary of the defining features of the five approaches most relevant to health inequalities, variation in definitions, how transportable an intervention informed by the different approaches might be to different contexts and the identified or implied logic for altering health inequalities. We discuss this in further depth below.

**Table 1:** Examples of interventions and policies adopting the five approaches

Approach	Examples*
Asset-based	Example 1: A community-based health promotion initiative to introduce traditional Indigenous games into schools and community groups. Identified community strengths through asset audits, enabled community control through community involvement in planning and decision-making and facilitated cultural exchanges.^S1^ Might be considered an intervention with potential to improve Indigenous health relative to other population groups. Example 2: Local neighbourhood project to improve physical activity opportunities for women in difficult life situations. A mapping exercise identified assets at individual (eg, participation by women from the intended population to help build a support network and recruit others), community (eg, sports club facilitating suitable space for sports activities) and infrastructure levels (eg, municipal office establishing a project office to legitimise the initiative).^S2^ Could therefore uplift health in women in difficult life situations relative to those who are not.
Place-based	Example 1: Co-developed green space interventions with local communities across several diverse parks in Los Angeles, USA, to improve both use of the parks and physical activity of the local communities.^S3^ Could therefore reduce geographical health inequalities by supporting health in these particular communities. Example 2: Best Start, an area-based intervention to improve breastfeeding rates in Victoria, Australia.^S4^ Created partnerships involving representatives from state and local government, non-government agencies and local communities and aimed to increase co-ordination, co-operation and linkages between services in neighbourhoods with high levels of social deprivation. Could reduce socio-economic inequalities in breastfeeding if increases rates in more deprived neighbourhoods.
Upstream	Example 1: Minimum unit pricing for alcohol: legislation set legal minimum price for alcohol, which makes cheaper alcohol typically consumed by the heaviest drinkers more expensive, therefore reducing alcohol-related health harms among those most at risk.^S5^ Example 2: B-Mincome: Barcelona minimum income pilot^S6^ Trial of providing vulnerable households a minimum income unconditionally without means testing.^S7^ Tackles a fundamental cause of health inequalities.
Systems	Example 1: WHO STOPS Childhood Obesity: a whole-of-community systems approach to address childhood obesity. Community and relevant organisations created a system map, used to identify and design actions to address childhood obesity. Engagement with community representatives with capacity to influence children’s food and activity environments to design actions to prevent childhood obesity, such as implementing water-only drink policies, and footpath and school drop-off points to facilitate walking to school.^S8^ Example 2: Healthy Families NZ: a systems-change intervention to prevent chronic diseases in 10 communities in Aotearoa New Zealand by strengthening community leadership and organisation. The communities have higher than average risks for preventable diseases and/or high levels of socio-economic deprivation. The intervention aimed to activate local leadership to influence change through ‘a dedicated systems thinking and acting health promotion workforce and activating local leadership to influence transformational change through local Strategic Leadership Groups’.^S9^
Proportionate universalism	Example 1: E-SEE Steps: parenting programme for parents of children under 2 years. One universal component, a parenting book, provided to all parents. Two further subsequent steps (group discussion sessions and at-home practice), determined by need, delivered as the children age.^S10^ The universal element could reduce stigma and increase engagement among higher need groups who were eligible for additional supports. Example 2: A city-wide housing-led renewal programme in Glasgow, Scotland, which allocated investment (for housing, eg, demolition, redesign, new build, housing improvements and social programmes, eg, employment support, debt management, playgrounds) according to need. Each area received investment, but this was higher in areas more disadvantaged in terms of markers of health, income and social factors.^S11^

The examples are the example studies’ authors’ language of the approaches used.

* References in online supplemental file.

**Table 2:** Key features of the five approaches*

Approach	Defining features relevant to health inequalities	Variations in definition	Transportability	Proposed or implied pathways to altered health inequalities
Asset-based	Inclusive, positive, involves collaborating with communities to find solutions.	Sometimes incorrectly described as an approach that identifies challenges and barriers (deficit-based interventions).^S12^	Focus on specific contexts makes individual assets-based interventions or programmes hard to scale up.^S12,S13^	Mechanisms to reducing health inequalities are not clearly articulated but are likely to include the targeting and co-production of solutions with community groups most likely to experience social disadvantage and ill health.^S14^
Place-based	Address the unique challenges and opportunities of particular places, recognising that geographic areas have different needs, sociodemographic profiles and health burdens.	Often (incorrectly) used to describe interventions which are geographically focused but pay limited attention to the features of that place or active community participation.	Similar to asset-based approaches, the focus on a specific place may limit transportability to other populations.	Potential to reduce inequalities by targeting areas of higher need and identifying locally tailored solutions. Can fail to incorporate higher-level policies needed to address the wider political and economic determinants of health. ^S15,S16^ Risk widening health inequalities, eg, regeneration changes residential demographics with more advantaged people moving into the area,^S17^ or if advantaged groups within the area benefit more.^S18^
Upstream and structural	Shifts focus from individually motivated behaviour change to the fundamental causes of social inequalities.	Original definition of the upstream metaphor (referring to prevention) is still in use. Often oversimplified to refer to a dichotomy of approaches classified as being either up or down stream.^S19,S20^	Transportability of interventions according to their placement in the ‘stream’ was not directly addressed in the literature although those which are further upstream may be more transportable.	Places less emphasis on individuals to make changes and creates more equal living environments. Unlikely to offer the sole solution—cannot stop everyone from falling into the river. Upstream / structural approaches have been criticised for disempowering individuals through downplaying the role of individual agency.^S21^
Systems	Comprehensive approach incorporating multilevel changes, adaptive, explores feedback loops and unintended consequences.	Imprecision in terminology leads to a lack of differentiation between systems thinking, systems methods and systems interventions.^S22^	Knowledge of and within systems is contextually bound.^S23^	Potential to address health inequalities is not clearly articulated^S24^ but speaks to the complexity of tackling health inequalities through identifying structural levers for change, intervening on multiple causes allowing for unintended consequences and seeking to change the beliefs and goals of the system (which could feature equity).
Proportionate universalism	Considered more ethical than targeted approaches, avoids stigma, benefits less advantaged groups, reduces waste of universal approaches.	Definition and operationalisation of need is ambiguous.^S25,S26^		Consequences for health inequalities dependent on how well need is defined and whether any additional or identified intervention is effective for the intended recipients.^S25^

* References in online supplemental file.

### Asset-based approaches

Asset-based approaches aim to identify and use existing community assets, or strengths, to enable people to have more control over their health and well-being. These are grounded in Antonovsky’s concept of salutogenesis,^15^ which focuses on positive health rather than ill health and disease, and where health can be co-created rather than fixed.^16^ The most common definition of a health asset is ‘any factor (or resource) which enhances the ability of individuals, groups, communities, populations, social systems and/or institutions to maintain health and wellbeing and to help to reduce inequalities’.^17^^, page 18^

Asset-based approaches seek to establish a common agenda, reframe thinking towards assets when establishing goals and outcomes, recognise existing and underused assets by mapping these with relevant communities at multiple levels,^16^ mobilise assets by proactively establishing connections to relevant stakeholders and co-produce new and required assets with the community.^18^

Much of the asset-based literature focuses on improving health and well-being rather than addressing health inequalities. However, they have been conceptualised as having the potential to address health inequalities^19^ ‘through strengthening social networks, empowering people to access and mobilise resources, and increasing their control over their own health and its wider determinants’”.^20^ The implied assumption is that asset-based approaches will reduce health inequalities by enhancing the things most likely to improve health, as well as supporting local resources and the local economy, in areas of highest need^20^ (inferring that need is akin to social disadvantage).

### Place-based approaches

Place-based approaches emerged in the 1960s as a way of addressing urban unrest and spatial concentrations of poverty.^21^ Place-based approaches target a specific geographical location and involve collaborative action across stakeholders including local government, business, third sector organisations and citizens to tackle the social determinants of health locally.^22^^, page 41^ There is variation in how they conceptualise place and conceive a place-based intervention, ranging from programmes that aim to alter the social, economic and physical environments of a *neighbourhood* to higher-level policy decision-making within national regions with devolved decision-making powers.^23^ Some place-based approaches differentiate between *compositional* factors (ie, the health behaviours and socio-economic characteristics of people in an area), *contextual* factors (ie, the physical and social environment of an area) and the *collective* (ie, an area’s social and cultural functions)^24^; however, these factors are increasingly seen as strongly interlinked.^25 26^

Place-based approaches are frequently cited as means to reducing health inequalities (eg, see Public Health England,^13^) by identifying and leveraging the factors most likely to improve health in areas with worse health outcomes relative to others. Our interpretation is therefore that their primary focus is reducing geographical inequalities, although a number of challenges have been identified, including the potential for creating stigma in these places, the bluntness of area-level deprivation for targeting socio-economically deprived individuals and the likelihood that those with the highest levels of resources within those areas will benefit most, if power relations are not also addressed.^27^

### Upstream approaches

The upstream-downstream metaphor was introduced to explain the challenges of dealing with the symptoms of a problem (pulling people from the river after they have fallen in) without addressing the underlying causes (the reason(s) why people fall in the river in the first place).^28^ Over time, the stream metaphor has become used to communicate approaches to tackling health inequalities.^29^ While most interventions cannot be easily dichotomised as being upstream or downstream (the stream after all is a continuum), the interventions furthest upstream are described as those that aim to address the underlying causes of health inequalities^30^—the unequal distribution of power, resources and wealth in the population. Also considered to be upstream are structural policies that aim to improve living and working environments, increase access to resources, reduce exposure to population-wide health harms and promote health-supporting societal norms.^3 31 32^

Upstream actions are often cited as being among the most promising for tackling health inequalities, because they tackle their fundamental causes. More downstream interventions tend to be individually focused, and therefore their success is more likely to rely on individual agency. While they can support health at the individual level, they can widen health inequalities, since people who have greater socio-economic resources are more likely to have the physical, emotional and financial resources to engage in and benefit from these interventions.^33^

### Systems approaches

Systems thinking is a perspective through which to understand and analyse any group of interconnecting parts that form a unified whole entity.^34^ Systems approaches were transferred from physical sciences following the UK government Foresight programme on obesity.^35^ Systems consist of elements (eg, individuals, households, organisations or countries), the interconnections and interactions between those elements and how these vary across space and time.^36^ Systems may be categorised as simple, complicated or complex^37^; it is the latter form that is most cited with respect to health inequalities. A key feature of complex systems is that their properties are more than the sum of the individual parts.^38^

A suite of systems methods is available to map and understand a system and consider potential consequences of hypothetical changes to that system.^39^ For example, the development of the intervention may be preceded by a mapping exercise, showing the pathways through which outcomes can arise, the influencers of those pathways and the connections between those influences.^40^ Changes can be unpredictable and may have different causal pathways leading to the same outcome.^41^ This process helps to identify different intervention points within the system and the possible consequences (both intended and unintended) of proposed interventions. For example, due to differential uptake of interventions across different socio-economic strata.^42^ During the design process, systems interventions will be refined in response to the mapping/modelling exercises to understand the potential scenarios produced by different options and engagement activities with communities and stakeholders.^43^

We refer to systems-based *approaches* as policies, interventions or programmes whose design has been shaped by systems thinking and methods, but we acknowledge that the term carries other meanings in the wider systems literature. A systems-based intervention is likely to involve multiple components, incorporate local contexts and react to changes that occur within the system(s) of interest as the intervention progresses.^43 44^ They have the potential to make changes that benefit multiple aspects of health and health determinants, by breaking the cycles that dampen the effects of interventions^45^ or reinforcing positive cycles of change over time.

Systems-based approaches are widely cited as a potential solution to reduce health inequalities.^45 46^ It stands to reason that their focus on complexity, multiple pathways and unintended consequences, for example, is likely to be beneficial, with this potential demonstrated by stakeholder work (eg, Van der Graaf).^47^ However, this requires consideration of these factors through a health inequality lens, not one that just considers overall health. This has not (to our knowledge) been fully articulated in the literature.

### Proportionate universalism

A proportionate universal policy or intervention is one that is applied universally, but with a scale and/or intensity proportionate to the level of disadvantage or need.^48^^, page 15^ Proportionate universalism entered the health inequalities field via the Marmot report in 2010^48^ and has been a widely cited solution since.^49^ This approach is more concerned with eligibility and dose of any given policy or programme than the policy or intervention lever.

There are some variations in how proportionate universalism is described and interpreted. For example, need can be conceptualised by the level of disadvantage, another health risk factor or current health needs. Degree of resource or support may comprise more of the same service or additional supports which can be tailored to individual needs. ‘Targeted universalism’ can be considered a synonym, while others refer to this specifically as a universal service which is tailored to maximise accessibility to less advantaged groups,^50^ who are most likely to require or benefit from that universal service.

The underlying logic for how proportionate universal approaches have the potential to reduce health inequalities is perhaps better articulated than some other approaches. By seeking to benefit everyone, but with increasing benefits according to need (often defined by level of social disadvantage), it aims to minimise stigma (and therefore maximise uptake in the highest need groups) and reduce health inequalities across the entire social gradient.^51^ However, the process for considering and operationalising proportionate universalism remains underdeveloped.

## A comparison of the different approaches with respect to health inequalities

For all approaches, the links to population health were well theorised although the assumed benefit to health inequalities was not always explicitly explained. One implied mechanism common to systems-based, place-based and assets-based approaches was community involvement. Co-producing policies and interventions with those they are designed to benefit, and the context in which they are being implemented, can help to identify the most fruitful policy levers, maximise their acceptability and reduce the possibility of inadvertently widening inequalities.^52^ Nevertheless, there was variation in the degree of community involvement, with this an inherent part of asset-based approaches, while a systems-based approach could acknowledge that communities are, and exist within, systems, without speaking with communities directly. Research evaluating community engagement practices and impacts on health inequalities has found that when objectives were not created by the communities, this can hinder community well-being and does not result in an anticipated reduction in inequalities.^53^

Another common thread was around target population. Place-based, assets-based and proportionate universal approaches are described (either directly or indirectly) as helping to tackle health inequalities through targeting places, communities or individuals experiencing the highest levels of need or social disadvantage. We argue, however, that this will depend on how well need or social disadvantage is defined and identified in the population. In addition, whether the target population groups (where relevant) are exposed to the intervention and, if active participation was required, whether the target population groups take up the intervention was rarely considered.

Upstream and proportionate universal approaches were highlighted as supporting uptake of interventions and policies among the groups most likely to suffer from the health consequences of social disadvantage. Proportionate universal interventions can do this by reducing stigma, while upstream interventions can minimise the need for individual agency, through producing cultural and societal changes and making living environments healthier for all.

The ‘level’ at which an intervention occurs impacts its potential to address health inequalities. Upstream approaches carry the advantage of incorporating the political or high-level structural changes that can address the root causes of health inequality for larger populations, though they may overlook the nuances of the contextual circumstances driving health inequalities within different communities or population subgroups. Place-based approaches tackle local issues, but their success may be undermined by national-level forces. A systems approach could—depending on where the boundaries of the system are drawn—incorporate both high-level structural components and ground-level context-specific components, maximising health benefits and reducing the potential for unintended consequences (such as a widening of health inequalities). Transportability is inevitably more limited for approaches centred on the needs of local communities. Place-based approaches posed a unique challenge whereby positive changes rooted in geographical locations can attract more advantaged population sub-groups and force out those who were intended to benefit. This could also apply to other approaches if they happen to be implemented within a specific location.

## Recommendations for articulating the anticipated and unanticipated consequences of interventions/policies on health inequalities

In box 1, we propose a series of questions that could be asked once a specific approach or intervention has been decided on or already implemented. These draw on common issues arising from our consideration of the five approaches and how they are conceived as impacting on health inequalities. They include a clearer definition of health inequalities, intended outcomes, expected mechanisms and key features including eligibility, uptake and effectiveness. These questions also chime with previous reviews and policy document analyses, which have, for example, identified a lack of specificity when defining and describing health inequalities.^9 14^ Our recommendations also draw heavily on a theoretical framework for considering the mechanisms through which inequalities might be altered^54^ and our previous work to anticipate impacts of hypothetical interventions and policies on health inequalities.^55^

Box 1 **Key questions to ask when designing or examining a policy/intervention which has the potential to alter health inequalities**

- Is the policy/intervention described as taking a particular approach*? How is this approach being defined?
- What is the policy/intervention aiming to change? What are the relevant health outcomes?
- What health inequalities is the intervention/policy intended to alter? Are there other axes of inequalitiesˆ that could also be relevant?
- How could the policy/intervention impact health inequalities? (Consider both positive and negative impacts.)
  - Altering the distribution of the relevant axis of inequality within the population (eg, redistributing income via taxation) (this is known as *social stratification*^54^).
  - Altering the distribution of health risk factors across the population, according to the relevant axis of inequality (eg, improving building quality standards for social housing to reduce damp) (*differential exposure*).
  - Altering the unequal health consequences of exposure to health risk factors (eg, introducing traffic control zones to reduce air pollution which can exacerbate the impacts of damp housing on respiratory health (*differential vulnerability*).
  - Altering the social consequences of ill health that are unequal across the relevant axis of inequality (eg, increasing Statutory Sick Pay) (*further social stratification*).
- Are there policy/intervention features that could alter health inequalities? (consider both positive and negative impacts)
  - Eligibility: Are there eligibility criteria and if so, are the eligibility criteria relevant to the identified axes of inequalities?
  - Uptake/exposure: How will exposure to, or uptake of, the intervention/policy occur? Could this be differential across axes of inequality?
  - Effectiveness: Of those who receive, or are exposed, to the policy/intervention, could some people benefit more than others?

*Could include proportionate universal, place-based, assets-based, systems-informed, upstream or any other categorisation. ˆFor example, income, education, neighbourhood deprivation, gender, ethnicity, disability, sexuality.

## Discussion

In order to address health inequalities, the WHO recommends paying attention to contextual specificities, cautioning that diverse social and political contexts will require diverse interventions, policies and approaches.^3^ Whitehead, in presenting a typology of actions, aimed to broaden understanding of the range of different levers available and discourage the tendency to focus on one type of intervention while neglecting others. The reasoning is that health inequalities are a complex problem and so no one type of action or intervention will suffice in isolation. Guiding principles such as those proposed by Davey *et al* help guide decision-makers towards the actions that could be most effective for reducing health inequalities.^8^ In this article, we have focused on several approaches that do not easily fit into existing typologies but nevertheless have been lauded as offering promise for reducing health inequalities. Rather than focussing on types of levers, as Whitehead’s typology does (eg, improving working and living conditions and macro-economic policies), the approaches we selected focused more on the process of designing interventions, in terms of their content, eligibility or roll-out and level of intervention within the system. We propose several questions that could be used to structure our considerations of how and why an intervention or policy may alter health inequalities. These are not intended to be used in isolation, but alongside other frameworks, including those mentioned above.

The five approaches were identified by the authors to be among the most commonly cited in recent literature and policy documents around tackling health inequalities. The disciplines of the author group include international relations, health law, system science, health economics, health geography, sociology, social policy, behavioural science, public health and epidemiology. However, we acknowledge the potential for disciplinary and contextual biases in the topic areas covered and welcome further debate in this area. There are other related approaches that we do not discuss here, such as life-course, universalism and behavioural approaches, which have valuable input for inequalities research and may be equally important. We also acknowledge that there are other approaches that are closely related to those that we discuss, for example, asset-based approaches share much in common with capability approaches, which focus on individual agency to support health and how this can be realised.^56 57^

Nevertheless, we have found that the logic behind the links between the approaches and health inequalities, or the mechanisms through which health inequalities are proposed to be influenced, was rarely well articulated. We therefore encourage researchers, practitioners and policy-makers to articulate this information when proposing policy recommendations, developing strategies or designing interventions. We suggest that this should involve a clear explanation of the approach(es) being developed or taken and the key proposed features, the types of health inequalities that are in focus, and the theory of change for how these health inequalities may (or may not) be reduced. We consider this an urgent but actionable priority, given the complexity of health inequalities and the lack of progress towards their reduction.

## Supplementary material

10.1136/jech-2025-224952 online supplemental file 1

## Data Availability

Data sharing not applicable as no datasets generated and/or analysed for this study.
