# Supplementary material for: Commonly cited approaches to reducing health inequalities: a call for more clarity around their definition and underlying assumptions
Source: J Epidemiol Community Health. 2025 Dec 9;80(4):e224952. doi: 10.1136/jech-2025-224952 (PMC13018810; doi:10.1136/jech-2025-224952)
Supplement: online supplemental file 1 [file jech-80-4-s001.docx]

**Commonly cited approaches to reducing health inequalities: a call for more clarity around their definition and underlying assumptions**

**Online supplementary file**

**References for Table 1 and Table 2**

S1. Parker E, Meiklejohn B, Patterson C, et al. Our games our health: a cultural asset for promoting health in Indigenous communities. *Health Promotion Journal of Australia* 2006;17(2):103-08. doi: https://doi.org/10.1071/HE06103

S2. Rütten A, Abu-Omar K, Frahsa A, et al. Assets for policy making in health promotion: Overcoming political barriers inhibiting women in difficult life situations to access sport facilities. *Social Science & Medicine* 2009;69(11):1667-73. doi: https://doi.org/10.1016/j.socscimed.2009.09.012

S3. Cohen DA, Han B, Derose KP, et al. Physical Activity in Parks: A Randomized Controlled Trial Using Community Engagement. *American Journal of Preventive Medicine* 2013;45(5):590-97. doi: https://doi.org/10.1016/j.amepre.2013.06.015

S4. Kelaher M, Dunt D, Feldman P, et al. The effect of an area-based intervention on breastfeeding rates in Victoria, Australia. *Health Policy* 2009;90(1):89-93. doi: https://doi.org/10.1016/j.healthpol.2008.08.004

S5. Wyper GMA, Mackay DF, Fraser C, et al. Evaluating the impact of alcohol minimum unit pricing on deaths and hospitalisations in Scotland: a controlled interrupted time series study. *The Lancet* 2023;401(10385):1361-70. doi: 10.1016/S0140-6736(23)00497-X

S6. Laín B, Torrens L. The minimum living wage initiative of Barcelona. *Transfer European Review of Labour and Research* 2019;25(3):386-91.

S7. García LR. The policy and political consequences of the B-Mincome pilot project. *European Journal of Social Security* 2022;24(3):213-29. doi: 10.1177/13882627221123347

S8. Allender S, Orellana L, Crooks N, et al. Four-Year Behavioral, Health-Related Quality of Life, and BMI Outcomes from a Cluster Randomized Whole of Systems Trial of Prevention Strategies for Childhood Obesity. *Obesity* 2021;29(6):1022-35. doi: https://doi.org/10.1002/oby.23130

S9. Matheson A, Walton M, Gray R, et al. Strengthening prevention in communities through systems change: lessons from the evaluation of Healthy Families NZ. *Health Promotion International* 2019;35(5):947-57. doi: 10.1093/heapro/daz092

S10. Berry V, Mitchell SB, Blower S, et al. Barriers and facilitators in the delivery of a proportionate universal parenting program model (E-SEE Steps) in community family services. *PLOS ONE* 2022;17(6):e0265946. doi: 10.1371/journal.pone.0265946

S11. Egan M, Kearns A, Katikireddi SV, et al. Proportionate universalism in practice? A quasi-experimental study (GoWell) of a UK neighbourhood renewal programme's impact on health inequalities. *Social Science and Medicine* 2016;152:41-49. doi: https://dx.doi.org/10.1016/j.socscimed.2016.01.026

S12. Martin-Kerry J, McLean J, Hopkins T, et al. Characterizing asset-based studies in public health: development of a framework. *Health Promotion International* 2023;38(2):daad015. doi: 10.1093/heapro/daad015

S13. McLean J. Positive Conversations, Meaningful Change: Learning from Animating Assets. Glasgow, UK: Glasgow Centre for Population Health and Scottish Community Development Centre, 2015.

S14. Roy MJ. The assets-based approach: furthering a neoliberal agenda or rediscovering the old public health? A critical examination of practitioner discourses. *Critical Public Health* 2017;27(4):455-64. doi: 10.1080/09581596.2016.1249826

S15. Bambra C. It’s the (political) economy. In: Bambra C, ed. Health Divides: Where You Live Can Kill You. Bristol: Bristol University Press 2016:137-82.

S16. McGowan VJ, Buckner S, Mead R, et al. Examining the effectiveness of place-based interventions to improve public health and reduce health inequalities: an umbrella review. *BMC Public Health* 2021;21(1):1888. doi: 10.1186/s12889-021-11852-z

S17. Moretti E. Place-based policies and geographical inequalities. *Oxford Open Economics* 2024;3(Supplement_1):i625-i33.

S18. McCartney G, Popay J. Are place-based approaches to reducing health inequalities a highway to success or a policy dead-end? *Journal of Critical Public Health* 2025; Advance online publication

S19. Krieger N. Proximal, Distal, and the Politics of Causation: What’s Level Got to Do With It? *American Journal of Public Health* 2008;98(2):221-30. doi: 10.2105/ajph.2007.111278

S20. Sniehotta FF, Araújo-Soares V, Brown J, et al. Complex systems and individual-level approaches to population health: a false dichotomy? *The Lancet Public Health* 2017;2(9):e396-e97. doi: 10.1016/S2468-2667(17)30167-6

S21. Lundberg O. Next steps in the development of the social determinants of health approach: the need for a new narrative. *Scandinavian Journal of Public Health* 2020;48(5):473-79. doi: 10.1177/1403494819894789

S22. Kwamie A, Ha S, Ghaffar A. Applied systems thinking: unlocking theory, evidence and practice for health policy and systems research. *Health Policy and Planning* 2021;36(10):1715-17. doi: 10.1093/heapol/czab062

S23. Jayasinghe S. Social determinants of health inequalities: towards a theoretical perspective using systems science. *International Journal for Equity in Health* 2015;14(1):71. doi: 10.1186/s12939-015-0205-8

S24. Trochim WM, Cabrera DA, Milstein B, et al. Practical challenges of systems thinking and modeling in public health. *Am J Public Health* 2006;96(3):538-46. doi: 10.2105/ajph.2005.066001 [published Online First: 2006/02/02]

S25. Francis-Oliviero F, Cambon L, Wittwer J, et al. Theoretical and practical challenges of proportionate universalism: a review. *Revista panamericana de salud publica = Pan American journal of public health* 2020;44:e110. doi: https://dx.doi.org/10.26633/RPSP.2020.110

S26. Carey G, Crammond B, De Leeuw E. Towards health equity: a framework for the application of proportionate universalism. *International journal for equity in health* 2015;14:81. doi: https://dx.doi.org/10.1186/s12939-015-0207-6
